# Assessment of the *In Vitro* and *In Vivo* Antitumor Activity of Hemocyanins from *Helix aspersa*, *Helix lucorum*, and *Rapana venosa* in a Graffi Myeloid Tumor Model

**DOI:** 10.3390/biomedicines11061545

**Published:** 2023-05-26

**Authors:** Ani Georgieva, Katerina Todorova, Ivan Iliev, Valeria Dilcheva, Ivelin Vladov, Svetlozara Petkova, Aleksandar Dolashki, Lyudmila Velkova, Pavlina Dolashka, Reneta Toshkova

**Affiliations:** 1 Institute of Experimental Morphology, Pathology and Anthropology with Museum, Bulgarian Academy of Sciences, Acad. G. Bonchev Str., Bl. 25, 1113 Sofia, Bulgaria; katerinagencheva@yahoo.com (K.T.); taparsky@abv.bg (I.I.); val_dilcheva@yahoo.com (V.D.); iepparazit@yahoo.com (I.V.); svetlozarapetkova@abv.bg (S.P.); reneta.toshkova@gmail.com (R.T.); 2 Institute of Organic Chemistry with Centre of Phytochemistry, Bulgarian Academy of Sciences, Acad. G. Bonchev Str., Bl. 9, 1113 Sofia, Bulgaria; adolashki@yahoo.com (A.D.); lyudmila_velkova@abv.bg (L.V.); pda54@abv.bg (P.D.)

**Keywords:** hemocyanins, Graffi myeloid tumor, antitumor activity, immunostimulating activity

## Abstract

Hemocyanins are oxygen-transporting glycoproteins in the hemolymph of some invertebrate species that attracted scientific interest as potential anticancer agents. The present study aims to assess the *in vitro* and *in vivo* anticancer activity of hemocyanins isolated from *Helix aspersa*, *Helix lucorum*, and *Rapana venosa* in the Graffi myeloid tumor model. The *in vitro* antitumor activity of the hemocyanins was determined by a MTT test and cytomorphological analysis by fluorescent and transmission electron microscopy. The *in vivo* effects of the hemocyanins were examined in hamsters transplanted with Graffi tumor. The serum antibody titers against the tested hemocyanins and tumor antigen were determined by ELISA. Histopathological assessment of the morphological features related to antitumor effect, immune system response, and toxicity in some internal organs was performed. The results of *in vitro* studies indicated that the tested hemocyanins induced significant antiproliferative and apoptogenic effects. The *in vivo* investigations demonstrated a protective antitumor effect, expressed in reduced transplantability, suppression of tumor growth and metastasis, reduced mortality, prolonged survival time, and absence of toxic side effects. The present study indicated that the antitumor activity of the studied hemocyanins was due to both immune stimulation and direct effects on the tumor cells, and they displayed their potential as therapeutic agents against hematological malignances.

## 1. Introduction

Hemocyanins (Hcs) are oxygen transporting glycoproteins in the hemolymph of some species in the phyla Arthropoda and Mollusca. These biomacromolecules have been extensively studied with respect to their chemical structure and biological activity [1,2,3]. Hcs belong to the type-3 copper protein family, which is characterized with the presence of an active site containing two copper atoms, each coordinated by three highly conserved histidine residues that are responsible for reversible binding of an oxygen molecule [4]. Despite the common physiological role, the chemical structure and glycosylation patterns of the hemocyanins from different arthropod and mollusc species demonstrate high heterogeneity [5]. Mollusc hemocyanins are usually organized as decamers or didecamers with molecular masses ranging from 4 to 8 MDa, forming hollow cylinders 35 nm in diameter that are readily observed by transmission electron microscopy [6]. The decamer is composed by self-assembly of ten subunits with molecular masses ranging from 350 kDa to 450 kDa depending on the species. The subunits contain seven to eight globular folded domains known as functional units (FUs), and each of them consists of α-helical domain that bears the active site and β-barrel domain [7]. The main recognized physiological functions of the hemocyanins are the binding, transportation, and delivery of oxygen from the respiratory organs to tissues. Aside from oxygen transport, Hcs are also known to participate in various physiological processes, such as hormone transport, osmoregulation, hemostasis, energy storage, moulting, and exoskeleton formation [4]. Substantial evidence gathered recently has revealed that hemocyanins act as a multifunctional protein associated with immune defense in invertebrates through their inducible phenoloxidase-like enzyme activity, and as precursors of antimicrobial peptides [3].

Over the past few decades, hemocyanins have been a focus of growing scientific interest due to their strong immunogenicity in mammals. Hemocyanins have found numerous applications in biomedicine and biotechnology as protein carriers to produce antibodies against small molecules, including drugs, hormones, peptides, tumor-specific antigens, as vaccine adjuvants, and as non-specific stimulators of the immune system in bladder cancer immunotherapy [8,9,10]. The immunostimulating properties of hemocyanins were attributed to their xenogenicity, large size, complex quaternary structure, and high glycan content [11,12]. Carbohydrate moieties of hemocyanins comprising up to 9% (*w*/*w*) of the molecules are considered responsible for their immunogenicity. Hemocyanin glycans are highly heterogeneous and primarily composed of high-mannose, complex, and hybrid type *N*-glycans that contribute to their structural stability and immunomodulatory properties in mammals [8]. The monosaccharide composition of mollusc glycans includes mannose, D-galactose, fucose, N-acetyl-D-galactosamine, and N-acetyl-glucosamine residues, of which mannose is the most abundant carbohydrate [13]. Research data have demonstrated that hemocyanin glycans act as multivalent ligands that bind with high affinities to innate immune receptors on the surface of the antigen-presenting cells (APCs), such as mannose receptors and Toll-like receptors, and triggers proinflammatory responses [11,14,15]. Hemocyanins are incorporated by APCs by both macropinocytosis and receptor-mediated endocytosis, then slowly processed, which facilitates a prolonged antigen presentation to T- or B-lymphocytes, and promotes potent cell-mediated and humoral immune responses [13]. Another potential mechanism that could contribute to the immunostimulatory properties of the hemocyanins is their ability to react with natural antibodies and to activate the classical pathway of the human complement system, with consequences in innate and adaptive immune response [16]. Moreover, hemocyanins have been found to stimulate the proliferation of natural killer (NK) cells, augment their cytotoxic activity, and enhance cytokines production [17].

Recently, different hemocyanins were found to suppress the proliferation and to alter the gene expression of human tumor cell lines [18,19].

The present study aims to assess the *in vitro* and *in vivo* antineoplastic potential of total hemocyanins isolated from *Helix aspersa* and *Helix lucorum* (HaH-total; HlH-total), their functional subunits (βc-HaH; α-HaH; βc-HlH; α-HlH), both subunits of the *Rapana venosa* hemocyanin (RvH I and RvH II), and *Helix aspersa* mucus (HaM) in the Graffi myeloid tumor model.

## 2. Materials and Methods

### 2.1. Isolation and Purification of Hemocyanins from H. lucorum, H. aspersa, and R. venosa and Mucus from H. aspersa

#### 2.1.1. Isolation of Native Hemocyanins and Their Structural Subunits

The hemolymph of *H. lucorum*, *H. aspersa*, and *R. venosa* was collected from a small cut of the foot muscles. In order to remove gross impurities, hemocytes, and other cells, the hemolymph was filtered and centrifuged at 10,000× *g* rpm for 20 min. The supernatant was concentrated by ultrafiltration on a membrane from 100 kDa (Millipore™ Ultrafiltration Membrane Filters, Regenerated cellulose, Burlington, MA, USA). The fraction with a molecular mass above 100 kDa, containing mainly hemocyanins, was subjected to ultracentrifugation at 22,000× *g* rpm with a Kontron-Hermle A8.24 rotor (CENTRIKON centrifuge) at 4 °C for 180 min and 210 min in order to obtain native hemocyanins. The stuructral subunits of HaH and HlH (α-HaH, βc-HaH, α-HlH and βc-HlH) were obtained from native hemocyanins dialized against 10 mM sodium acetate buffer at 4 °C. The α- and βc-subunits were separated and purified by gel filtration chromatography as previously described [20,21]. The subunits of *R. venosa* hemocyanin (RvH I and RvH II) were isolated after dialysis against 0.13 M glycine/NaOH buffer, pH 9.6 for 12 h. Separation of the two subunits was performed by ion-exchange chromatography using a 16/10 Q Sepharose^®^ High Performance column equilibrated with 50 mM Tris/HCl buffer, 10 mM EDTA, pH 8.5, with a linear gradient of 0.0–0.5 M NaCl by a FPLC system.

#### 2.1.2. Isolation of *H. aspersa* Mucus

The mucus was collected from *H. aspersa* snails, grown in Bulgarian eco-farms by a patented technology [22]. The obtained crude mucus extract was purified by several steps of centrifugation and filtration, as previously described [22,23].

### 2.2. In Vitro Experiments

#### 2.2.1. Cell Cultures and Cultivation

Graffi tumor cells were isolated from the tumor tissue under aseptic conditions, as described earlier [24]. Graffi myeloid tumor cells were grown as monolayers in 25 cm^2^ tissue culture flasks in a RPMI-1640 medium (Gibco BRL, Grand Island, NY, USA) containing 10% fetal bovine serum, 2 mM L-glutamine, 100 U mL^−1^ penicillin, and 100 µg mL^−1^ streptomycin. The cultures were maintained at 37 °C in a humidified atmosphere with 5% CO_2_. All culture reagents were purchased from Gibco/BRL (Grand Island, NY, USA).

#### 2.2.2. MTT Assay

The *in vitro* antiproliferative activity of the hemocyanins was studied on Graffi myeloid tumor cells using the standard MTT assay [25]. The trypsinized tumor cells (1 × 10^5^ cells/mL) in RPMI-1640, containing 10% FBS, were plated (100 μL/well) in 96-well flat-bottomed microplates (Orange Scientific, Braine-l’Alleud, Belgium) and allowed to adhere for 24 h. The cells were then treated with hemocyanins at concentrations of 31.25, 62.5, 125, 250, 500, and 1000 µg/mL (six wells per concentration) for 72 h. Untreated tumor cells were used as negative controls. The standard cytostatic doxorubicin (DOX) was applied to Graffi tumor cells under the same culture conditions and time of treatment as the positive control. At the end of treatment, the culture medium was discarded, the cells were rinsed with PBS, and 100 μL of MTT (3-(4,5-dimethylthyazol-2-yl)-2,5-diphenyl tetrazolium bromide) solution (0.5 mg/mL) was added to each well. The plates were further incubated for 3 h, at 37.5 °C in a CO_2_ incubator. Formazan crystals were dissolved by adding 100 μL/well of lysing ethanol/DMSO (1:1 *v*/*v*) solution and the absorption was measured using a microplate reader (TECAN, Sunrise TM, Groedig/Salzburg, Austria) at 570 nm.

#### 2.2.3. Acridine Orange/Ethidium Bromide (AO/EB) Dual Staining

Analysis of tumor cell morphology and the apoptosis-inducing ability of the tested hemocyanins were examined by AO/EB double staining, according to standard procedures [26] with a minor modification [27]. Acridine orange is permeable to viable cells and can directly intercalate into DNA emitting green fluorescence. Ethidium bromide is a dye that stains cells with increased membrane permeability (dead and late apoptotic cells) and emits red-orange fluorescence. Graffi cells were grown on glass coverslips placed in 24-well plates for 24 h. The cells were than exposed to α-HaH, βc-HlH, and RvH II at concentrations approximating the IC_50_ values determined by the MTT assay. After 24 h of incubation, the glass lamellas were rinsed with phosphate buffered saline (PBS) and stained with equal volumes of fluorescent dyes AO (10 μg/mL) and EB (10 μg/mL). Stained cells were immediately examined under a fluorescent microscope (Leica DM 500B, Wetzlar, Germany).

#### 2.2.4. DAPI Staining

The nuclear morphology of the hemocyanin-treated cells was examined by fluorescent microscopy after staining with 4′,6-Diamidine-2′-phenylindole dihydrochloride (DAPI) according to the manufacturer instructions. Graffi tumor cells were seeded and cultured with and without the hemocyanins, as described for the AO/EB staining. After 24 h of treatment, the glass lamellas with adherent tumor cells were washed, fixed with methanol, and stained for 15 min with 1 µg/mL DAPI in methanol at room temperature in the dark. Stained cells were mounted with glycerol on microscope slides and analyzed by a fluorescent microscope (Leica DM 500B, Wetzlar, Germany).

#### 2.2.5. Transmission Electron Microscopy (TEM)

Ultrastructural changes of Graffi cells treated with hemocyanins at concentrations approximately equal to their IC_50_ values were analyzed by transmission electron microscopy. For this purpose, control and hemocyanin-treated cells were fixed for 1 h with 2.5% glutaraldehyde in a 0.1 M phosphate buffer (pH 7.3), postfixed for 2 h in 1% OsO_4_, and dehydrated and embedded in Durcupan ACM Fluka. Ultra-thin sections were prepared by Reichert Ultramicrotome and stained with 2% uranyl acetate and 2% lead citrate. The preparations were imaged by an Opton transmission electron microscope.

### 2.3. In Vivo Experiments

#### 2.3.1. Animals and Animal Care

In the experiments, 2–3 month old female and male Golden Syrian hamsters weighing about 100 g, obtained from the breeding base of the National specialized hospital for active treatment of hematological diseases Sofia, were used. After an acclimatization period in the Vivarium of IEMPAM-BAS, the hamsters were individually housed in plastic cages and raised with free access to food and water. All procedures were carried out in accordance with the national regulation No 20/01.11.2012 regarding laboratory animals and animal welfare, the European directive 2010/63/EU of the European Parliament, and of the Council of 22 September 2010 on the protection of animals used for scientific purposes. The study was approved by the Bulgarian Agency for Food Safety, approval number 282, from 24 September 2020.

The Graffi experimental tumor is a transplantable, rapidly growing myeloid tumor with a high degree of malignancy. It is characterized by 100% occurrence, 100% mortality, and no spontaneous regression. The tumor was originally induced in newborn hamsters by the Graffi murine leukemia virus and was adapted to grow in a solid form in mature animals. The tumor was maintained by the subcutaneous (s.c.) injection of 0.5 mL of tumor cell suspension (2 × 10^4^ viable cells) into the dorsal region. This experimental tumor is a reliable model for studying the anticancer effects of natural and synthetic compounds, providing accurate and reproducible results [28].

#### 2.3.2. *In Vivo* Antitumor Activity Studies

The *in vivo* antitumor activity of the hemocyanins and their subunits was assessed in female and male hamsters. The hamsters were randomly divided into ten experimental groups (Control-TBH, HaH-total, βc-HaH, α-HaH, HaM, HlH-total, βc-HlH, α-HlH, RvH I, and RvH II) of seven animals each. Each hamster from the experimental groups were injected s.c. in the back area three times at intervals of 7 days with 0.2 mL of the vaccine preparation, containing 40 µg of the respective hemocyanin sample and aluminum hydroxide as the adjuvant. The control group of hamsters received a treatment with PBS. Seven days after the last immunization, experimental and control hamsters were subcutaneously transplanted with 2 × 10^4^ viable Graffi tumor cells in their back area. Two hamsters from the separate groups were sacrificed by decapitation under medical sedation at day 10 after tumor transplantation. The blood and tissue samples obtained were used for hematological, serological, and histopathological analyses. *In vivo* antitumor activity was also evaluated by measurement of the parameters of tumor growth.

#### 2.3.3. Hematological Analysis

Peripheral blood from the control and tumor-bearing hamsters was collected. Blood samples (20 μL) from each hamster were subjected to analysis using an automatic hematology analyzer (BC-2800 Vet, Mindray, Shenzhen, China), and nineteen blood parameters were measured. Hematometric indices including the quantitative ratio of leukocytes and lymphocytes (WBC/Ly), neutrophils and lymphocytes (N/Ly), and platelets/lymphocytes (PLT/Ly) were calculated.

#### 2.3.4. Serological Analysis

An indirect ELISA was performed in 96-well, flat-bottom microplates (Greiner Bio-One, Kremsmunster, Austria). The wells were coated overnight at 4 °C with antigen (1 µg/100 µL/well) in PBS (pH 7.2). After washing, 100 µL of a blocking solution (3% dry milk in PBS) was added to each plate well for 1 h at room temperature. 100 µL of the diluted serum sample was added to each well and the plates were then incubated for 2 h at room temperature. After washing the plates, 100 µL of Protein A-HRP (Sigma, Saint Louis, MO, USA) diluted 1:40,000 was added and incubated for 90 min at room temperature. After the final washings, 100 µL of the peroxidase substrate o-Phenylenediamine (Sigma, Saint Louis, MO, USA) was added and incubated for 20 min at room temperature. The absorbance was measured at 492 nm with a microplate reader. Each serum sample was tested in four replicates. All washings of the plates were performed three times with 0.05% Tween 20 in PBS. Results were expressed as an Optical Density (OD) index. 

#### 2.3.5. Assessment of Biometric Parameters of Tumor Growth

*In vivo* antitumor activity was examined and the following parameters were evaluated: tumor transplantability (%) was determined as a ratio between the number of hamsters with palpable (detectable) tumors and the total number of hamsters in the experimental group; the tumor size (mm) for each animal was determined with calipers by measuring two perpendicular diameters of the tumor nodule at regular intervals until day 30 after tumor inoculation; the mean tumor size (mm) was calculated for each experimental group; lethality (%), which is the proportion of dead hamsters on the respective day of observation after tumor cell inoculation, as compared to the total number of animals in the group; the survival rate was followed for each group until the end of the experiments (50th day after tumor transplantation).

#### 2.3.6. Histopathological Analysis

The animals were observed after inoculation until they were euthanized. Two animals were randomly selected from each group for harvesting of tumor, liver, kidney, and spleen tissue. The harvested tissues were stored in 10% buffered formalin for pathological analysis. The tissues were dehydrated, paraffin embedded, and sectioned into 4 µm thickness. The sections from each tissue sample were stained with hematoxylin and eosin (H&E) and analyzed using the microscope Leica DM 5000 B.

### 2.4. Statistical Analysis

Statistical analysis was performed by one-way ANOVA followed by Bonferroni’s post hoc test (GraphPad Software Inc., San Diego, CA, USA). Values of *p* < 0.05 were considered statistically significant. The concentrations inducing 50% inhibition of the cell growth (IC_50_ values) were determined by nonlinear regression (curve fit) analysis (GraphPad Prism). The OD index was calculated as the ratio of measured absorbance values of the test serum samples and absorbance value of negative control serum samples. The values were presented as the mean value of OD index and Standard Error of the Mean (SEM).

## 3. Results

### 3.1. Isolation of Hemocyanins and Their Isoforms

The native intact HlH as well as HaH, in contrast to many molluscan hemocyanins, are presented in the hemolymph as two α-HlH—isoforms (αD and αN) and one βc-HlH isoform and respectively two α-HaH and one βc-HaH [18,20,21]. In this study, the tested hemocyanins—total hemocyanins from garden snails *H. lucorum* and *H. aspersa* (HlH-total and HaH-total), their isoforms (subunits βc-HlH, and α_(N+D)-_HlH and respectively βc-HaH, α_(N+D)-_HaH), both subunits of *R. venosa* hemocyanin (RvH I and RvH II), and *H. aspersa* mucus—were isolated and purified as previously described [18,20,21]. The total hemocyanins were isolated from the hemolymph of snails *H. aspersa*, *H. lucorum*, and *R. venosa* after ultrafiltration, using membrane 100 kDa and following ultracentrifugation. After dissociation of native RvH by dialysis against a dissociating buffer (0.13 M glycine/NaOH (pH 9.6)) two structural subunits, RvH I and RvH II with a Mw 400 kDa and 420 kDa, respectively, were isolated on an ion-exchange chromatography. The subunits βc-HlH and βc-HaH were purified by gel filtration chromatography on a Sepharose 6B column and eluted with buffer 50 mM Tris-HCl, pH 7.5. The isoforms α_(N+D)_-HlH and α_(N+D)_-HaH also were purified by gel filtration chromatography [20,21]. The molecular masses and purity of the hemocyanins and their subunits were confirmed by 8% native PAGE [20].

### 3.2. Effects of the Hemocyanins on the Viability and Proliferative Activity of Graffi Tumor Cells In Vitro

**Figure 1:**
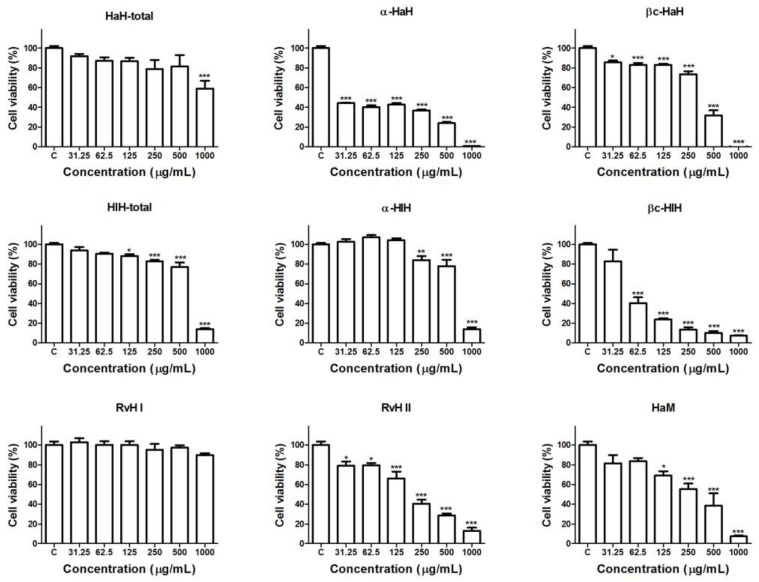
Antiproliferative and cytotoxic effects of 72 h treatment with hemocyanins isolated from *H. aspersa*, *H. lucorum*, and *R. venosa* on Graffi tumor cells. HaH-total and HlH-total—total hemocyanins isolated from *H. aspersa* and *H. lucorum*; α-HaH, βc-HaH, α-HlH and βc-HlH—structural subunits of the *H. aspersa* and *H. lucorum* hemocyanins; HaM—*H. aspersa* mucus; RvH I and RvH II structural subunits of the *R. venosa* hemocyanin. Data are presented as mean ± SD; * *p* < 0.05, ** *p* < 0.01, *** *p* < 0.001, compared to the negative control.

Hemocyanins isolated from *H. aspersa*, *H. lucorum*, and *R. venosa* were tested for antiproliferative and cytotoxic activity on Graffi myeloid tumor cells by a MTT test after 72 h of exposure (Figure 1).

The results of the MTT analysis demonstrated a statistically significant and concentration-dependent reduction of the viability of the tumor cells after treatment with most of the tested hemocyanin samples. The treatment with RvH I subunit of *R. venosa* hemocyanin did not significantly affect the viability of the tumor cells and the total hemocyanin of *H. aspersa* reduced the cell viability only at the higher tested concentration. The α-subunit of *H. aspersa* was the most active of the studied hemocyanins. The cytotoxic effects of all tested samples were significantly lower as compared to the positive control drug DOX. The concentrations of the tested and control compounds that inhibit the cell growth of Graffi cells by 50% (IC_50_) are presented at Table 1.

Based on the calculated IC_50_ values, the most active samples of hemocyanins from each mollusc species was determined and used for further morphological studies for elucidation of the mechanisms of their antitumor effects and the type of cell death induced in the Graffi tumor cells. 

### 3.3. Dual Staining of Graffi Tumor Cells with Acridine Orange/Ethidium Bromide

**Figure 2:**
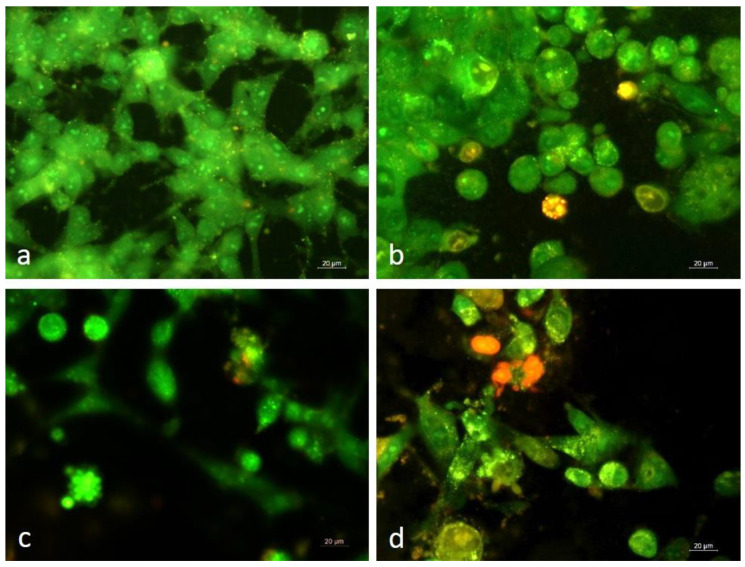
Cytomorphological alterations of Graffi myeloid tumor cells stained with acridine orange/ethidium bromide after 24 h treatment with hemocyanins isolated from *H. aspersa*, *H. lucorum*, and *R. venosa.* (**a**) control untreated cells; (**b**) cells treated with 30 µg/mL subunit α-HaH; (**c**) cells treated with 60 µg/mL subunit βc-HlH; (**d**) cells treated with 200 µg/mL subunit RvH II.

The apoptogenic potential of α-HaH subunit, βc-HlH subunit, and RvH II subunit was examined by dual staining with acridine orange/ethidium bromide. For this purpose, the tumor cells were exposed to the subunit βc-HlH and subunit RvH II in concentrations equal to IC_50_ values obtained by MTT assay and the subunit α-HaH was applied in the lower concentration used for the cell viability test. Morphological alterations induced by the tested hemocyanins in the tumor cells after 24 h of treatment were registered by fluorescent microscopy (Figure 2). 

The fluorescent microscopy study illustrated that control cells were with normal morphology and monolayer growth, characteristic of the Graffi tumor cell line and uniformly green stained. The cells treated with α-HaH subunit, βc-HlH subunit, and RvH II subunit revealed distinct morphological changes. Impaired monolayer growth and cells with signs of early (intense green fluorescence and condensation of chromatin in the form of dense green areas) and late apoptosis (red-orange stained cells with aggregated chromatin and fragmented nuclei) were observed in treated cell cultures.

### 3.4. DAPI Staining of Graffi Tumor Cells

**Figure 3:**
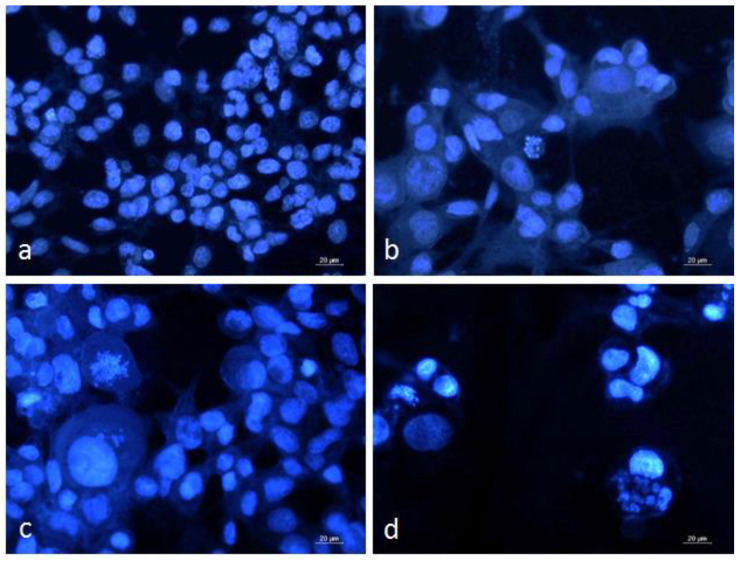
Fluorescence microscopic images of DAPI stained Graffi myeloid tumor cells after 24 h treatment with hemocyanins isolated from *H. aspersa*, *H. lucorum*, and *R. venosa*. (**a**) control untreated cells; (**b**) cells treated with 30 µg/mL subunit α-HaH; (**c**) cells treated with 60 µg/mL subunit βc-HlH; (**d**) cells treated with 200 µg/mL subunit RvH II.

The alterations in nuclear morphology induced by α-HaH, βc-HlH, and RvH II subunits in the Graffi cells were analyzed by fluorescent microscopy after DAPI staining (Figure 3). 

The control cells were uniform in shape and size with homogenous blue staining. The hemocyanin-treated cells demonstrated marked nuclear morphological abnormalities, such as anisokaryosis and nuclear pleomorphism. Some of the cells had intense blue staining due to the chromatin condensation characteristic for early apoptosis and others displayed late apoptotic changes like fragmentation of the nucleus and formation of apoptotic bodies. 

The results of the cytomorphological analysis indicated that the *in vitro* antitumor effects of the tested hemocyanins are associated with induction of apoptosis.

### 3.5. Transmission Electron Microscopy

**Figure 4:**
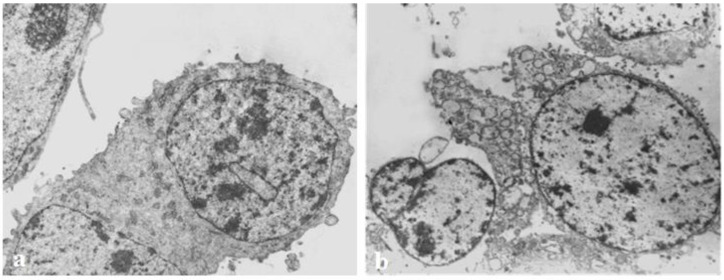
Transmission electron microscopy images of the morphological alterations induced in Graffi tumor cells by hemocyanins isolated from *H. aspersa*. (**a**) control cells; (**b**) cells treated with 30 µg/mL subunit α-HaH for 24 h. Instrumental magnification 300 K.

The ultrastructural alterations in the hemocyanin-treated Graffi tumor cells were further analyzed by transmission electron microscopy (Figure 4).

The electron microscopy of cells treated with hemocyanins exhibited lightened nuclei with individual condensates of chromatin, focal expansion of perinuclear spaces, and strong vacuolization in the cytoplasm and cell organelles, as well as budding with the formation of apoptotic bodies with vacuoles.

### 3.6. Hematological Studies

**Figure 5:**
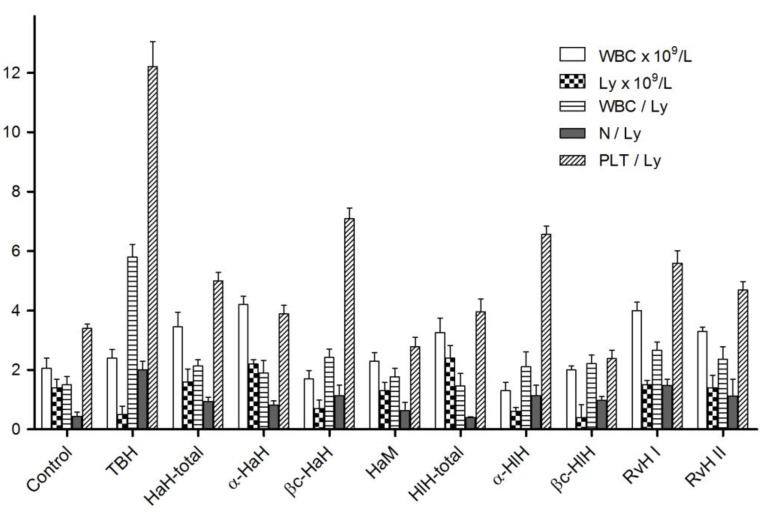
Effects of the hemocyanins treatment on hematological parameters and hematometric indices of Graffi tumor-bearing hamsters. TBH—tumor-bearing hamsters; HaH-total and HlH-total—total hemocyanins isolated from *H. aspersa* and *H. lucorum*; α-HaH, βc-HaH, α-HlH, and βc-HlH—structural subunits of the *H. aspersa* and *H. lucorum* hemocyanins; HaM—*H. aspersa* mucus; RvH I and RvH II—structural subunits of the *R. venosa* hemocyanin.

The effects of experimental immunotherapy with hemocyanins on the white blood cells count (WBC), lymphocyte count (Ly) and white blood cells/lymphocytes ratio (WBC/Ly), neutrophils/lymphocytes ratio (N/Ly), and platelets/lymphocytes (PTL/Ly) were studied in blood samples taken 10 days after tumor cell transplantation (Figure 5). 

As demonstrated in the figure, WBC counts increased, lymphocyte counts decreased, and the WBC/Ly, N/Ly, and PLT/Ly ratios significantly increased in tumor-bearing hamsters, compared to healthy animals. Significant improvement of the WBC/Ly and N/Ly indexes was found in all groups of experimental animals treated with hemocyanins and their subunits. PLT/Ly ratios were also markedly ameliorated compared to TBH, especially in α-HaH, HaM, HlH-total, and βc-HlH treated groups.

### 3.7. Serological Studies

**Figure 6:**
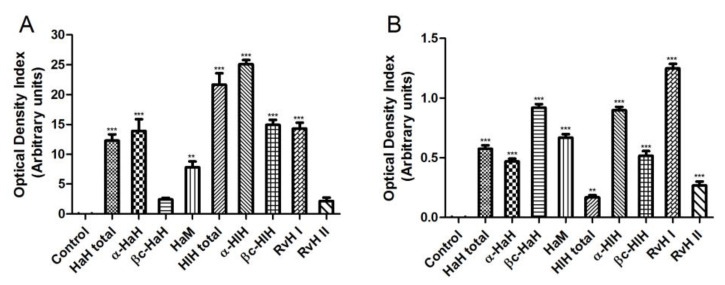
ELISA analysis of serum antibody titers of hamsters with Graffi myeloid tumor treated with hemocyanins. (**A**) hemocyanin antigens; (**B**) tumor antigen. HaH-total and HlH-total—total hemocyanins isolated from *H. aspersa* and *H. lucorum*; α-HaH, βc-HaH, α-HlH, and βc-HlH—structural subunits of the *H. aspersa* and *H. lucorum* hemocyanins; HaM—*H. aspersa* mucus; RvH I and RvH II—structural subunits of the *R. venosa* hemocyanin. ** *p* < 0.01, *** *p* < 0.001, compared to the control.

Assessment of the immunostimulating effects of treatment with different hemocyanins was performed by the indirect ELISA method (Figure 6A). Sera were tested at 1/1000 dilution. The highest titers of specific antibodies were observed in sera obtained from experimental animals treated with HlH-total and α-HlH (about 25 arbitrary units). In addition, we performed an indirect ELISA to determine cross-reactivity between the specific immune sera against the investigated hemocyanins and the tumor antigen of the Graffi myeloid tumor (Figure 6B). Serum samples were tested at a 1/50 dilution. All hemocyanin samples induced a statistically significant increase (*p* < 0.001) in the antibody titers against the tumor-specific antigen. The serum titers of antibodies against the Graffi tumor antigen were highest in experimental animals treated with βc-HaH, α-HlH, and RvH I.

### 3.8. Assessment of Biometric Parameters of Tumor Growth

**Figure 7:**
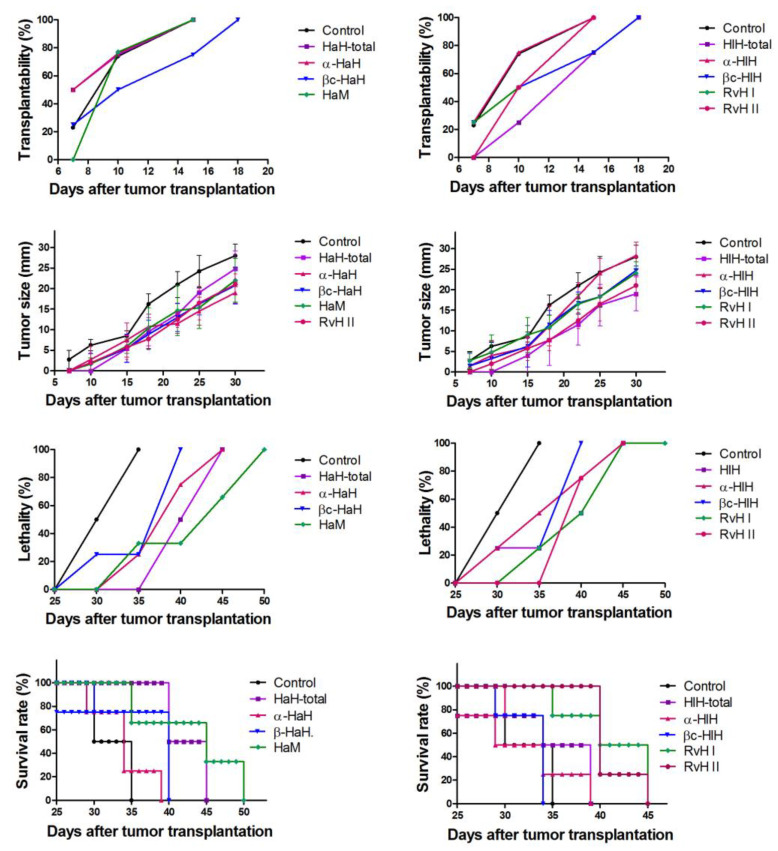
Effects of the treatment with hemocyanins on some biometric parameters of Graffi tumor growth. HaH-total and HlH-total—total hemocyanins isolated from *H. aspersa* and *H. lucorum*; α-HaH, βc-HaH, α-HlH, and βc-HlH—structural subunits of the *H. aspersa* and *H. lucorum* hemocyanins; HaM—*H. aspersa* mucus; RvH I and RvH II—structural subunits of the *R. venosa* hemocyanin.

Hemocyanin treatment induced marked changes of the measured parameters of tumor growth (Figure 7). The effects on the transplantablility were most pronounced in experimental groups treated with βc-HaH, HlH-total and βc-HaH. In these groups, appearance of tumors in 100% of the experimental animals was established at the 18th day after tumor transplantation, while in the untreated control the tumors appeared in all animals at the 15th day. The subunits α-HaH, βc-HaH, RvH II, and HlH-total induced stronger inhibition of the tumor growth and demonstrated the lowest values of measured tumor size. The mucus of *H. aspersa* and RvH II induced the most significant reduction of lethality and an increase in survival rate.

### 3.9. Histopathological Analysis

**Figure 8:**
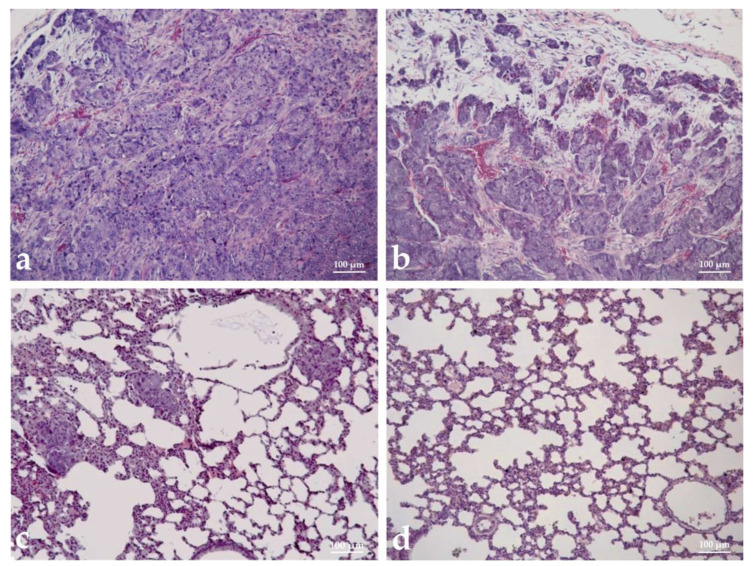
Histopathological analysis of malignancy and metastatic potential of Graffi myeloid tumor in hamsters treated with α-subunit of hemocyanin isolated from *H. aspersa*. (**a**) tumor tissue of control tumor-bearing hamster (TBH); (**b**) tumor tissue from α-HaH-treated TBH; (**c**) lung from control TBH; (**d**) lung from α-HaH-treated TBH.

Histopathological analysis was performed to assess the malignancy, metastatic potential, immune system response, and toxicity of tested hemocyanins in tumor-bearing hamsters (TBH). The tumor tissue sampling in immunized animals displayed a higher degree of cell differentiation, with a well-defined capsule and stroma. In contrast, in non-immunized animals, poorly differentiated cells, anisokaryosis, and anisocytosis were observed and the stroma was in minimal quantities (Figure 8a,b). 

**Figure 9:**
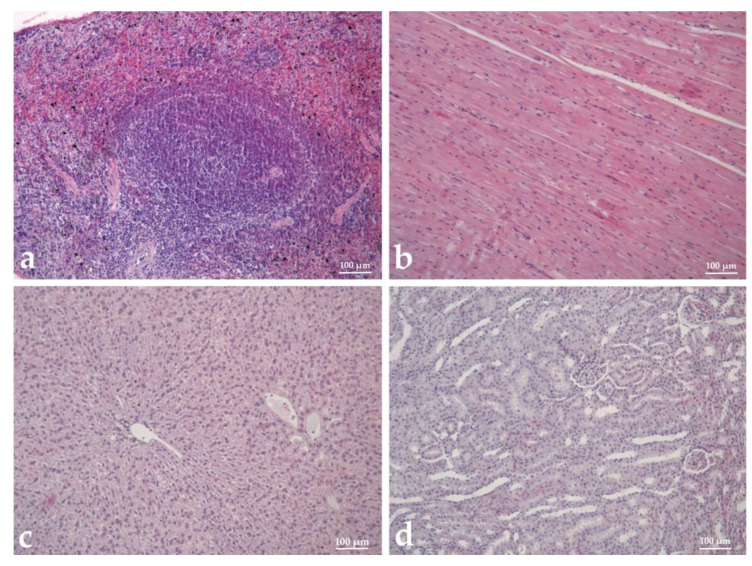
Histopathological analysis of immune system response and toxicity of α-subunit of hemocyanin isolated from *H. aspersa*. (**a**) spleen from α-HaH-treated tumor-bearing hamster (TBH); (**b**) heart from α-HaH-treated TBH; (**c**) liver from α-HaH-treated TBH; (**d**) kidney from α-HaH-treated TBH.

Regarding the metastatic potential of the myeloid tumor, no metastases were found in the organs of hemocyanin-treated animals (Figure 8d), except for single basophilic cells in atrial tissues of individual hamsters immunized with βc-HlH, while in control untreated animals, metastatic nodules were found in the lungs (Figure 8c). A morphological analysis of the organs related to the immune reaction was performed on experimental animals (Figure 9a). In the groups treated with HlH and α-HlH, a paracortical type of lymphoid tissue hyperplasia was observed in the lymph nodes, involving preference of T cell stimulation. In the spleen, follicular hyperplasia was observed and the white pulp predominated as a ratio to the red pulp. The degree of reactive hyperplasia in the spleen and lymph nodes exceeded that observed in the controls. No deviations from the norm were detected in the liver or kidneys of the treated groups. The tested hemocyanins at the used concentrations and schemes of treatment did not cause pathomorphological changes, indicating toxic damage of the visceral organs of the treated hamsters. Cardiotoxicity, hepatotoxicity, and nephrotoxicity were not observed (Figure 8b–d) and the spleen and pancreas also did not display pathohistological changes associated with hemocyanin treatment.

## 4. Discussion

Hemocyanins are major extracellular oxygen-transporting proteins in the hemolymph of many mollusc species, which have been found to exert strong immunogenicity in mammals and are extensively studied as potential anticancer therapeutics. Among them, the keyhole limpet hemocyanin (KLH) isolated from the marine gastropod *Megathura crenulata* is the most thoroughly investigated with respect to immunostimulating and anticancer properties. In clinical studies, a marked reduction of recurrence rate of superficial urinary bladder cancer after treatment with KLH has been established [13]. This hemocyanin has found a broad spectrum of biomedical applications as an adjuvant and protein carrier for anticancer vaccines [29,30]. However, the limited geographic distribution of *Megathura crenulata* has prompted the efforts to find hemocyanins of other species with similar biological activities. In our study, the antitumor and immunostimulating potential of hemocyanins isolated from *H. aspersa*, *H. lucorum*, and *R. venosa* were assessed. Hemocyanins isolated from *H. lucorum* and *R. venosa* were previously found to exhibit anticancer effects on human bladder carcinoma cells [19,31,32,33]. Moreover, hemocyanins derived from *H. lucorum* displayed a higher inhibitory effect on cancer cell proliferation compared to KLH [31]. The mechanism of *in vitro* antitumor effects of HlH and RvH has been found to include the downregulation of genes related to cell proliferation and upregulation of genes involved in apoptosis as well as in immune system activation [18]. Other studies indicated that the antitumor effects of these hemocyanins are not limited to bladder cancer cells. A decrease in the viability of ovarian, prostate, and glioma cancer cells, acute monocytic leukemia, and Burkitt’s lymphoma cells has also been reported [34]. 

Most of the studies have been focused on cancer cell lines of epithelial cell origin and there is limited data about the antitumor activity of hemocyanins against hematological malignancies. For this reason, the Graffi myeloid tumor was chosen as an experimental model in the present study. The results indicated that the studied hemocyanins induced a statistically significant reduction in the viability and proliferation activity of *in vitro* cultured Graffi myeloid cells. In a previous study, the antiproliferative and cytotoxic effects of the same hemocyanin samples were examined on the non-cancerous cell line of mice embryo fibroblasts Balb/c 3T3, clone A31 [20]. The IC_50_ values of the tested compounds determined by the MTT test on the non-tumorigenic cells were higher than the values established in the Graffi tumor cells, thus demonstrating the selectivity of the cytotoxic action of the hemocyanins with respect to tumor cells. Among the nine tested samples, α-HaH induced the strongest cytotoxic effects. This finding is in accordance with previously published data indicating that individual structural subunits display higher cytotoxic activity than the native form of hemocyanins. 

The subunits of *H. lucorum* and *H. aspersa* hemocyanins have been found to induce stronger inhibition of the tumor growth of bladder carcinoma CAL-29 cells and colorectal carcinoma cell HT-29 than the intact biomolecules [19,20]. Numerous studies have suggested that the oligosaccharide components of the hemocyanins are mainly responsible for their anticancer effects [3,8,35]. It could be hypothesized that the stronger tumor inhibitory activity is due to the specific carbohydrate moieties that are more easily accessible in the separated structural subunits than in the native oligomeric glycoprotein complex. Our previous study revealed a highly heterogeneous mixture of N-glycans corresponding to complex and high-mannose types in RvH and HlH [12]. The glycans of HlH mainly contain a terminal MeHex residue; in some cases, even two, three, or four of these residues occur. Several carbohydrate chains in β-HlH are core-fucosylated without Xyl and also possess a high degree of methylation. The structural studies of the isoforms of RvH demonstrated the presence of unusual N-glycan structures with an internal fucose residue (β1-2)-connecting GalNAc and a hexuronic acid, as well as sulfated mannose, methylated GlcNAc, and methylated galactose [36,37]. The identified N-glycans from high-mannose and complex type in RvH and HlH could play an important role in the interaction between these molecules and the target tumor cells. The investigated samples of hemocyanins were previously tested for antitumor activity on the HT-29 cell line [20]. The results of the present study indicated that myeloid tumor cells appeared to be more sensitive to the cytotoxic and antiproliferative effects of the hemocyanins than the colorectal carcinoma cells.

The hemocyanins that displayed the highest *in vitro* antitumor activity on the Graffi cells were further analyzed by fluorescent and transmission electron microscopy. Cytomorphological studies of hemocyanin-treated tumor cells revealed typical features of apoptosis, such as chromatin condensation, karyopiknosis, nucleus fragmentation, and formation of apoptotic bodies. Previously reported data indicated an increase in early and late apoptosis in the MCF-7 breast carcinoma cell line and significantly reduced cellular proliferation *in vitro* in melanoma cells, via early apoptotic pathways after treatment with KLH [38,39]. Hemocyanin from *Litopenaeus vannamei* (LvH) induced typical morphological features of apoptosis and an apoptotic ladder was detected in HeLa cells [40]. Apoptosis induction by *H. aspersa*, *H. lucorum*, and *R. venosa* hemocyanins was also reported in urinary bladder and colon carcinoma cells [18,20,33,34]. Promising and antitumor activity against human mammary carcinoma cell lines of different molecular subtypes, mediated by the induction of apoptosis has recently been reported for the two structural subunits of RvH and a fraction with Mw between 50 and 100 kDa isolated from the hemolymph of the marine snail *R. venosa* [41]. These findings indicate that despite the diversity in the chemical structure of the hemocyanins derived from different species, the mechanism of their antitumor activity involves apoptosis induction.

Further evidence for the antitumor properties of different hemocyanins was obtained by *in vivo* studies in different cancer models. Hemocyanins isolated from the gastropod *Concholepas concholepas* induced immunotherapeutic effects, decreased tumor growth, and prolonged survival without any toxic side effects in a murine bladder cancer model [42]. Strong *in vivo* anticancer and antiproliferative effects of hemocyanins from *Rapana thomasiana* and *Helix pomatia* were expressed in a murine model of colon carcinoma. Immunization with these hemocyanins stimulated a humoral anticancer response, decreased tumor growth and metastasis appearance, and prolonged the survival of treated animals [43]. Hemocyanins from *Fissurella latimarginata* stimulated the humoral immune response and displayed potent antitumor activity, delaying tumor growth and increasing survival in mouse models of melanoma [6]. Significant immunogenicity of hemocyanins from *C. concholepas* and *F. latimarginata* reduced tumor development and increased overall survival was reported in mouse models of oral squamous cell carcinoma [44]. The present study is the first report that analyses the potential antineoplastic effects of hemocyanins derived from *H. aspersa*, *H. lucorum*, and *R. venosa* in a virus-induced myeloid cancer model.

Oncological diseases are usually accompanied by hematological abnormalities. The changes in basic hematological indicators are used as a diagnostic and prognostic tool in patients with malignant diseases. In the present study, the WBC counts measured in the healthy, tumor-bearing, and hemocyanin-treated animals were within the reference range for this species. Slightly increased WBC counts indicating immune response activation were found in the groups treated with HaH-total, α-HaH, HlH-total, and RvH I. Platelets have been found to play a key role in the progression of the malignant neoplasms and contribute to local tumor growth, spread, and metastasis. Platelet counts and the platelet-lymphocyte ratio are used as a prognostic indicator for the course of tumor diseases [45,46]. Increased platelet count is associated with more aggressive tumor behavior, increased risk of thrombosis, and higher mortality [45]. The platelet numbers in the tumor-bearing hamsters displayed a marked increase, which was significantly ameliorated in the animals subjected to hemocyanin immunization.

Another important hematological index evaluated in our study is the neutrophils to lymphocytes ratio (NLR), which is considered a valuable predictor of cancer survival. For example, an NLR value ≤ 2.0 is a good prognostic marker for longer survival in breast cancer patients, while an NLR value ≥ 5.0 is considered a poor prognostic marker for several cancer types, including breast cancer, gastric cancer, and others [46]. A marked decrease of the NLR ratio in all hemocyanin-treated groups, compared to non-treated tumor bearing controls, was found. These findings were in agreement with the results obtained from analysis of the biometric parameters that demonstrated a protective antitumor effect, expressed in reduction of transplantability, inhibition of tumor growth, reduced mortality, and prolonged survival. 

Additional data on the antineoplastic activity of the studied samples were obtained from the performed histopathological analysis. Tumor tissues of immunized animals displayed higher cell differentiation and a well-defined capsule and stroma, in contrast to the poorly differentiated cells with pronounced anisokaryosis and anisocytosis observed in the untreated tumor-bearing controls. 

Another important finding was the lack of metastases in the hemocyanin-immunized animals, whereas the control untreated animals displayed multiple metastatic nodules in the lung tissues. Moreover, no signs of inflammation, necrosis, calcification, or other pathology in the tissues of the hemocyanin-immunized animals were found, except for the tumors that possessed some of these characteristics. The observations of the immune organs (spleens and lymph nodes) displayed clear signs of activation in the compartments responsible for the maintenance of naive antibody-producing B cells as well as T cells, especially in animals treated with HlH and α-HlH. The immunostimulating effect of different hemocyanins was also confirmed by an indirect ELISA method. A statistically significant increase (*p* < 0.001) was found in the titer of antibodies against the Graffi tumor-specific antigen in the serum of the immunized hamsters, compared to the non-immunized controls. The obtained results indicate the potential of the investigated hemocyanins for cancer immunotherapy.

## 5. Conclusions

The present study analyzes the potential of hemocyanins isolated from *H. aspersa*, *H. lucorum*, and *R. venosa* as anticancer therapeutics in a myeloid cancer model. The tested samples displayed significant antiproliferative and pro-apoptotic effects on *in vitro* cultured Graffi myeloid tumor cells. The *in vivo* experiments in a myeloid tumor model indicate that these bioactive compounds exert distinct immunostimulating and antitumor effects without any histopathological signs of toxicity. These findings reveal the investigated hemocyanins as promising candidates for immunotherapy of hematological cancer diseases.

## Figures and Tables

**Table 1:** Inhibitory concentrations (IC_50_) of the bioactive compounds isolated from *H. aspersa*, *H. lucorum*, and *R. venosa* and the positive control substance doxorubicin (DOX), determined by MTT assay on Graffi myeloid tumor cells.

Hemocyanins.	IC_50_ Values (µg/mL)
HaH-total	>1000
HaH-α	<30
HaH-βc	340.2
HaM	259.5
HlH-total	650.9
HlH-α	659.6
HlH-βc	61.1
RvH I	>1000
RvH II	194.1
DOX	0.2

## Data Availability

All data necessary to understand or reproduce this study are included in the manuscript.

## References

[1] Kato S., Matsui T., Gatsogiannis C., Tanaka Y. (2018). Molluscan hemocyanin: Structure, evolution, and physiology. Biophys. Rev..

[2] Coates C.J., Decker H. (2017). Immunological properties of oxygen-transport proteins: Hemoglobin, hemocyanin and hemerythrin. Cell. Mol. Life Sci..

[3] Coates C.J., Costa-Paiva E.M. (2020). Multifunctional roles of hemocyanins. Subcell Biochem..

[4] Coates C.J., Nairn J. (2014). Diverse immune functions of hemocyanins. Dev. Comp. Immunol..

[5] Van Holde K.E., Miller K.I., Decker H. (2001). Hemocyanins and invertebrate evolution. J. Biol. Chem..

[6] Arancibia S., Espinoza C., Salazar F., Del Campo M., Tampe R., Zhong T.Y., De Ioannes P., Moltedo B., Ferreira J., Lavelle E.C. (2014). A novel immunomodulatory hemocyanin from the limpet *Fissurella latimarginata* promotes potent anti-tumor activity in melanoma. PLoS ONE.

[7] González A., Nova E., Del Campo M., Manubens A., De Ioannes A., Ferreira J., Becker M.I. (2017). The oxygen-binding properties of hemocyanin from the mollusk *Concholepas concholepas*. Biochim. Biophys. Acta Proteins Proteom..

[8] Salazar M.L., Jiménez J.M., Villar J., Rivera M., Báez M., Manubens A., Becker M.I. (2019). N-Glycosylation of mollusk hemocyanins contributes to their structural stability and immunomodulatory properties in mammals. J. Biol. Chem..

[9] Becker M.I., Arancibia S., Salazar F., Del Campo M., De Ioannes A. (2014). Mollusk Hemocyanins as natural immunostimulants in biomedical applications. Immune Response Activation.

[10] Lammers R.J., Witjes W.P., Janzing-Pastors M.H., Caris C.T., Witjes J.A. (2012). Intracutaneous and intravesical immunotherapy with keyhole limpet hemocyanin compared with intravesical mitomycin in patients with non-muscle-invasive bladder cancer: Results from a prospective randomized phase III trial. J. Clin. Oncol..

[11] Jiménez J.M., Salazar M.L., Arancibia S., Villar J., Salazar F., Brown G.D., Lavelle E.C., Martínez-Pomares L., Ortiz-Quintero J., Lavandero S. (2019). TLR4, but neither Dectin-1 nor Dectin-2, participates in the mollusk hemocyanin-induced proinflammatory effects in antigen-presenting cells from mammals. Front. Immunol..

[12] Velkova L., Dolashka P., Van Beeumen J., Devreese B. (2017). N-glycan structures of β-HlH subunit of Helix lucorum hemocyanin. Carbohydr. Res..

[13] Arancibia S., Salazar F., Becker M.I. (2012). Hemocyanins in the immunotherapy of superficial bladder cancer. Bladder Cancer—From Basic to Robotic Surgery.

[14] Zhong T.Y., Arancibia S., Born R., Tampe R., Villar J., Del Campo M., Manubens A., Becker M.I. (2016). Hemocyanins stimulate innate immunity by inducing different temporal patterns of proinflammatory cytokine expression in macrophages. J. Immunol..

[15] Villar J., Salazar M.L., Jiménez J.M., Campo M.D., Manubens A., Gleisner M.A., Ávalos I., Salazar-Onfray F., Salazar F., Mitchell D.A. (2021). C-type lectin receptors MR and DC-SIGN are involved in recognition of hemocyanins, shaping their immunostimulatory effects on human dendritic cells. Eur. J. Immunol..

[16] Pizarro-Bauerle J., Maldonado I., Sosoniuk-Roche E., Vallejos G., López M.N., Salazar-Onfray F., Aguilar-Guzmán L., Valck C., Ferreira A., Becker M.I. (2017). Molluskan hemocyanins activate the classical pathway of the human complement system through natural antibodies. Front. Immunol..

[17] Sarker M.M.R., Zhong M. (2014). Keyhole limpet hemocyanin augmented the killing activity, cytokine production and proliferation of NK cells, and inhibited the proliferation of Meth A sarcoma cells *in vitro*. Indian J. Pharmacol..

[18] Antonova O., Yossifova L., Staneva R., Stevanovic S., Dolashka P., Toncheva D. (2015). Changes in the gene expression profile of the bladder cancer cell lines after treatment with *Helix lucorum* and *Rapana venosa* hemocyanin. J. Buon..

[19] Dolashki A., Dolashka P., Stenzl A., Stevanovic S., Aicher W.K., Velkova L., Velikova R., Voelter W. (2019). Antitumour activity of Helix hemocyanin against bladder carcinoma permanent cell lines. Biotechnol. Biotechnol. Equip..

[20] Georgieva A., Todorova K., Iliev I., Dilcheva V., Vladov I., Petkova S., Toshkova R., Velkova L., Dolashki A., Dolashka P. (2020). Hemocyanins from Helix and Rapana snails exhibit *in vitro* antitumor effects in human colorectal adenocarcinoma. Biomedicines.

[21] Velkova L., Dimitrov I., Schwarz H., Stevanovic S., Voelter W., Salvato B., Dolashka-Angelova P. (2010). Structure of hemocyanin from garden snail *Helix lucorum*. Comp. Biochem. Physiol. B Biochem. Mol. Biol..

[22] Dolashki A., Velkova L., Daskalova E., Zheleva N., Topalova Y., Atanasov V., Voelter W., Dolashka P. (2020). Antimicrobial Activities of Different Fractions from Mucus of the Garden Snail *Cornu aspersum*. Biomedicines.

[23] Vassilev N.G., Simova S.D., Dangalov M., Velkova L., Atanasov V., Dolashki A., Dolashka P. (2020). An 1H NMR- and MS-Based Study of Metabolites Profiling of Garden Snail Helix aspersa Mucus. Metabolites.

[24] Gardeva E., Toshkova R., Yossifova L., Minkova K., Ivanova N., Gigova L. (2014). Antitumor activity of C-phycocyanin from *Arthronema africanum* (Cyanophyceae). Braz. Arch. Biol. Technol..

[25] Mossmann T. (1983). Rapid colorimetric assay for cellular growth and survival: Application to proliferation and cytotoxicity assays. J. Immunol. Meths..

[26] Wahab I., Abdul A., Alzubairi A., Elhassan M., Mohan S. (2009). *In vitro* ultramorphological assessment of apoptosis induced by zerumbone on (HeLa). J. Biomed. Biotechnol..

[27] Ignatova M.G., Manolova N.E., Toshkova R.A., Rashkov I.B., Gardeva E.G., Yossifova L.S., Alexandrov M.T. (2010). Electrospun nanofibrous mats containing quaternized chitosan and polylactide with *in vitro* antitumor activity against HeLa cells. Biomacromolecules.

[28] Toshkova R., Manolova N., Gardeva E., Ignatova M., Yossifova L., Rashkov I., Alexandrov M. (2010). Antitumor activity of quaternized chitosan-based electrospun implants against Graffi myeloid tumor. Int. J. Pharm..

[29] Gilewski T.A., Ragupathi G., Dickler M., Powell S., Bhuta S., Panageas K., Koganty R.R., Chin-Eng J., Hudis C., Norton L. (2007). Immunization of high-risk breast cancer patients with clustered sTn-KLH conjugate plus the immunologic adjuvant QS-21. Clin. Cancer Res..

[30] McFadden D.W., Riggs D.R., Jackson B.J., Vona-Davis L. (2003). Keyhole limpet hemocyanin, a novel immune stimulant with promising anticancer activity in Barrett’s esophageal adenocarcinoma. Am. J. Surg..

[31] Boyanova O., Dolashka P., Toncheva D., Rammensee H.-G., Stevanovic S. (2013). *In vitro* Effect of Molluscan Hemocyanins on CAL-29 and T-24 Bladder Cancer Cell Lines. Biomed. Rep..

[32] Georgieva A., Todorova K., Iliev I., Dilcheva V., Vladov I., Petkova S., Toshkova R., Velkova L., Atanasov V., Dolashki A. (2021). *In vitro* antitumour activity of hemocyanins isolated from *Helix Aspersa* and *Helix Lucorum* in human bladder carcinoma cells. Comptes Rendus L’académie Bulg. Sci..

[33] Dolashki A., Antonova O., Velkova L., Kaynarov D., Voelter W., Dolashka P. (2022). Selective cytotoxicity and changes in protein expression of T24 bladder carcinoma permanent cell line after treatment with hemocyanins. Curr. Med. Chem..

[34] Antonova O., Toncheva D., Rammensee H.G., Floetenmeyer M., Stevanovic S., Dolashka P. (2014). *In vitro* Antiproliferative Effect of Helix aspersa Hemocyanin on Multiple Malignant Cell Lines. Z. Naturforsch. C.

[35] Dolashka P., Dolashki A., Velkova L., Stevanovic S., Molin L., Traldi P., Velikova R., Voelter W. (2015). Bioactive compounds isolated from garden snails. J. BioSci. Biotechnol..

[36] Dolashka-Angelova P., Beck A., Dolashki A., Beltramini M., Stevanovic S., Salvato B., Voelter W. (2003). Characterization of the carbohydrate moieties of the functional unit RvH(1)-a of *Rapana venosa* haemocyanin using HPLC/electrospray ionization MS and glycosidase digestion. Biochem. J..

[37] Dolashka P., Velkova L., Shishkov S., Kostova K., Dolashki A., Dimitrov I., Atanasov B., Devreese B., Voelter W., Van Beeumen J. (2010). Glycan structures and antiviral effect of the structural subunit RvH2 of Rapana hemocyanin. Carbohydr. Res..

[38] Riggs D.R., Jackson B.J., Vona-Davis L., Nigam A., McFadden D.W. (2005). *In vitro* effects of keyhole limpet hemocyanin in breast and pancreatic cancer in regards to cell growth, cytokine production, and apoptosis. Am. J. Surg..

[39] Somasundar P., Riggs D.R., Jackson B.J., McFadden D.W. (2005). Inhibition of melanoma growth by hemocyanin occurs via early apoptotic pathways. Am. J. Surg..

[40] Zheng L., Zhao X., Zhang P., Chen C., Liu S., Huang R., Zhong M., Wei C., Zhang Y. (2016). Hemocyanin from shrimp *Litopenaeus vannamei* has antiproliferative effect against HeLa cell *in vitro*. PLoS ONE.

[41] Petrova M., Vlahova Z., Schröder M., Todorova J., Tzintzarov A., Gospodinov A., Velkova L., Kaynarov D., Dolashki A., Dolashka P. (2023). Antitumor Activity of Bioactive Compounds from *Rapana venosa* against Human Breast Cell Lines. Pharmaceuticals.

[42] Moltedo B., Faunes F., Haussmann D., De Ioannes P., De Ioannes A.E., Puente J., Becker M.I. (2006). Immunotherapeutic effect of Concholepas hemocyanin in the murine bladder cancer model: Evidence for conserved antitumor properties among hemocyanins. J. Urol..

[43] Gesheva V., Chausheva S., Mihaylova N., Manoylov I., Doumanova L., Idakieva K., Tchorbanov A. (2014). Anti-cancer properties of gastropodan hemocyanins in murine model of colon carcinoma. BMC Immunol..

[44] Mora Román J.J., Del Campo M., Villar J., Paolini F., Curzio G., Venuti A., Jara L., Ferreira J., Murgas P., Lladser A. (2019). Immunotherapeutic potential of mollusk hemocyanins in combination with human vaccine adjuvants in murine models of oral cancer. J. Immunol. Res..

[45] Giannakeas V., Kotsopoulos J., Brooks J.D., Cheung M.C., Rosella L., Lipscombe L., Akbari M.R., Austin P.C., Narod S.A. (2022). Platelet count and survival after cancer. Cancers.

[46] Zhang L.X., Wei Z.J., Xu A.M., Zang J.H. (2018). Can the neutrophil-lymphocyte ratio and platelet-lymphocyte ratio be beneficial in predicting lymph node metastasis and promising prognostic markers of gastric cancer patients? Tumor maker retrospective study. Int. J Sur..

